# Management of the thrombotic risk associated with COVID-19: guidance for the hemostasis laboratory

**DOI:** 10.1186/s12959-020-00230-1

**Published:** 2020-09-07

**Authors:** M. Hardy, T. Lecompte, J. Douxfils, S. Lessire, J. M. Dogné, B. Chatelain, S. Testa, I. Gouin-Thibault, Y. Gruel, R. L. Medcalf, H. ten Cate, G. Lippi, F. Mullier

**Affiliations:** 1 Université catholique de Louvain, CHU UCL Namur, Namur Thrombosis and Hemostasis Center (NTHC), Hematology Laboratory, Yvoir, Belgium; 2 Université catholique de Louvain, CHU UCL Namur, Namur Thrombosis and Hemostasis Center (NTHC), Anesthesiology Department, Yvoir, Belgium; 3 Département de Médecine, Hôpitaux Universitaires de Genève, service d’angiologie et d’hémostase et Faculté de Médecine Geneva Platelet Group (GpG), Université de Genève, Geneva, Suisse Switzerland; 4 Pharmacy Department, University of Namur, Namur Thrombosis and Hemostasis Center (NTHC), Namur, Belgium; 5 Qualiblood s.a, Namur, Belgium; 6 Haemostasis and Thrombosis Center, Cremona Hospital, Cremona, Italy; 7 Département d’Hématologie Biologique, INSERM, CIC 1414 (Centre d’Investigation Clinique de Rennes), Université de Rennes, CHU de Rennes, Rennes, France; 8 Laboratoire d’Hématologie-Hémostase, CHRU de Tours, Hôpital Trousseau, Tours, France; 9 Australian Centre for Blood Diseases, Monash University, Melbourne, Victoria Australia; 10 Department of Internal Medicine, Cardiovascular Research Institute (CARIM), Maastricht University Medical Center, Maastricht, the Netherlands; 11 Section of Clinical Biochemistry, University of Verona, Verona, Italy

**Keywords:** Thrombosis, D-dimers, Anticoagulation, COVID-19, Coagulopathy, Hemostasis, Fibrinolysis, Heparin, Thrombin generation, Viscoelastic tests

## Abstract

Coronavirus disease 2019 (COVID-19) is associated with extreme inflammatory response, disordered hemostasis and high thrombotic risk. A high incidence of thromboembolic events has been reported despite thromboprophylaxis, raising the question of a more effective anticoagulation. First-line hemostasis tests such as activated partial thromboplastin time, prothrombin time, fibrinogen and D-dimers are proposed for assessing thrombotic risk and monitoring hemostasis, but are vulnerable to many drawbacks affecting their reliability and clinical relevance. Specialized hemostasis-related tests (soluble fibrin complexes, tests assessing fibrinolytic capacity, viscoelastic tests, thrombin generation) may have an interest to assess the thrombotic risk associated with COVID-19. Another challenge for the hemostasis laboratory is the monitoring of heparin treatment, especially unfractionated heparin in the setting of an extreme inflammatory response. This review aimed at evaluating the role of hemostasis tests in the management of COVID-19 and discussing their main limitations.

## Introduction

Since the beginning of the coronavirus infectious disease 2019 (COVID-19) pandemic in December 2019, increasing data have supported a major thrombotic risk, which could explain a substantial part of morbidity and mortality associated with this infection. The first observations in China reported a marked increase in plasma D-dimers, associated with unfavorable prognosis and enhanced thrombotic risk [1, 2]. A recent meta-analysis demonstrated an association between several inflammatory biomarkers (such as C-reactive protein (CRP), procalcitonin, interleukin (IL-6) and ferritin) with COVID-19 severity [3]. There is a cross-talk between inflammation and coagulation as inflammation leads to coagulation activation, and coagulation also affects inflammatory activity [4, 5]. Although heparin at prophylactic doses appears to be effective in reducing mortality in severe COVID-19 patients [6], several studies reported a considerably high incidence of venous and even arterial thromboembolic events (deep vein thrombosis, pulmonary embolism or in situ thrombosis in the pulmonary arteries; arterial thromboses in the systemic circulation) despite thromboprophylaxis, thus raising the issue of increasing anticoagulant doses [7–14]. This approach has been suggested by several groups of experts for patients at higher thrombotic risk [15–19]. However, this suggestion has not been endorsed by other expert groups or societies [20–23]. Prospective trials are currently in progress to try to answer this question.

The features and specificities of hemostasis disorders associated with COVID-19 are partially known only. In general, severe pulmonary inflammation (acute respiratory distress syndrome (ARDS)) is associated with a significant thrombotic risk, the main mechanisms of which include (i) local expression of tissue factor (TF) on mononuclear cells, its accumulation and release following endothelial damage, which subsequently initiates coagulation initiation and thrombin generation, (ii) as well as inhibition of fibrinolysis in response to the cytokine storm (TNFα, IL-1, IL-6) [24–26]. IL-1 and TNF-α are also the main cytokines responsible for the impairment of endogenous anticoagulant pathways [27]. Increase in von Willebrand factor (vWF), FVIII and fibrinogen plasma levels was also observed [28]. Regarding fibrinolysis, an initial and transient increase of tissue plasminogen activator (tPA) has been described in severe acute inflammatory states, rapidly followed by a sustained release of PAI-1 by the endothelium [28], the extend of the latter being associated with a worse outcome [29]. Accordingly, t-PA and PAI-1 levels were increased at ICU admission in ARDS patients in a recent study, whether COVID-19 or not [30]. Levels of thrombin activatable fibrinolysis inhibitor (TAFI) and protein C inhibitor were also found to be significantly elevated in the bronchoalveolar fluid of patients with interstitial lung disease when compared to healthy controls, which could also add to the hypofibrinolysis observed [31, 32]. However, this hypothesis seems at odds with the marked increase in plasma D-dimers observed in COVID-19 patients. This needs to be further investigated in patients with COVID-19.

Other biomarkers of in vivo activation of blood coagulation such as soluble fibrin complexes may also be informative in assessing the thrombotic risk, but their usefulness remains to be established. In this article, we will be using the term ‘D-dimers’ to designate the (soluble) fibrin degradation products containing the motif ‘D-dimers’ and that of soluble fibrin complexes (SFC) to designate complexes containing one or more fibrin monomers associated with two fibrinogen molecules or with fibrinogen (or even fibrin) degradation products (Table 1).

**Table 1:** Definition of D-dimers, fibrin monomers, fibrinogen degradation products and fibrin degradation products

Entity	Definition	Antigenic definition (what is recognized by the antibodies of the immunoassay – immunogen that was used to obtain the antibodies in the animal)
**Degradation products containing the ‘D-dimers’ motif (fibrin degradation products)**	Soluble molecular assemblies produced by the action of plasmin on polymerized fibrin stabilized by factor XIIIa, hence comprising the ‘D-dimers’ motif	Two ‘D’ nodules of two adjacent fibrin monomers in the fibrin network, covalently linked by factor XIIIa
**Soluble fibrin complexes**	Complexes formed of at least one fibrin monomer associated with fibrinogen molecules, but also fibrinogen or even fibrin degradation products – those complexes are soluble in plasma	desAA-fibrin (fibrinogen from which fibrinopeptides A have been removed)
**Fibrinogen degradation products**	Molecules produced by the action of plasmin on fibrinogen (when there systemic fibrinolysis occurs)	Nodule ‘D’ or ‘E’ of fibrinogen: present on fibrinogen and their degradation products; fibrinogen must therefore be eliminated beforehand so as not to be measured

Endothelial activation by inflammatory cytokines (with secretion of von Willebrand factor, factor VIII and PAI-1) and dysfunction could also contribute to the prothrombotic state observed in COVID-19 patients [33]. Previous studies identified possible direct infection of endothelial cells by SARS-CoV-2 with endothelialitis [34] and increased circulatory endothelial cells, an indicator of endothelial damage [35]. As in many severe inflammatory states, thrombotic microangiopathy (TMA) may develop secondary to acquired deficiency of ADAMTS13 and subsequent increase in large von Willebrand factor multimers [27, 36]. In COVID-19, signs of localized pulmonary TMA were observed with typical microvascular platelet-rich thrombi identified in small vessels of the lungs and other organs. Von Willebrand factor antigen and activity were reported to be elevated and ADAMTS13 levels to be decreased, although remaining higher than 10% [27, 36, 37]. However, schistocytes were not identified on blood smears, signs of hemolysis were absent and platelet count was higher than expected in this condition [27].

With limited solid data available, many questions remain unanswered regarding underlying mechanisms but also stratification and management of thrombotic risk, including the level of anticoagulation and the duration of treatment. Some first-line hemostasis tests (i.e., activated partial thromboplastin time (APTT), prothrombin time (PT), fibrinogen, D-dimers, platelet count) are easy to perform, widespread and relatively inexpensive, but have also significant drawbacks, which should be clearly considered in guiding clinical management. The reliability of D-dimers, especially at very high values, as well as the respective role of anti-Xa activity and APTT for monitoring therapy with unfractionated heparin (UFH), will be discussed in the following parts of this article. The indications and limitations of tests available in the hemostasis laboratory are summarized in Table 2.

**Table 2:** Indications and limitations of tests available in the hemostasis laboratory

	Main Limitations	Indications in COVID-19					
		Evaluation of the thrombotic risk	Screening of thromboembolic events	Prognosis: disease severity	Diagnosis of DIC	Detection of HIT	Monitoring of unfractionated heparin
**Platelet count**	Many causes of thrombocytopenia in the critically ill patient			X	X	X	
**APTT**	Major influence of preanalytical step					X (*)	X
	Differences of APTT reagents in their sensitivity to unfractionated heparin, lupus anticoagulant and inflammatory syndrome						
**Prothrombin Time**	Influence of the preanalytical step				X		
	Influence of fibrinogen level						
**Fibrinogen (Clauss)**	Lack of sensitivity for the diagnosis of DIC (mostly in infectious/inflammatory setting)	X		X	X		
	Possibility of interference of direct thrombin inhibitors with some reagents						
**D-dimers**	Decreased analytical performances in high D-dimers values	X	X	X	X		
	Production dependent on the fibrinolytic activity						
	Cut-offs non commutable between reagents						
**Soluble fibrin complexes (AKA ‘Fibrin monomers’)**	Preliminary validation only		X		X		
	Not evaluated in COVID-19						
	Cut-offs non commutable between the reagents						
**Fibrinolysis capacity tests**	Fibrinolysis is assessed in non-physiological conditions	X		X			
	Large array of methods						
	Not evaluated in COVID-19						
**Anti-Xa activity**	Inter-reagent variability						X
	Influence of presence/absence of exogenous AT in the reagent						
	Expensive						
	Only available 24 h a day 7 days a week in a fraction centers						
**Viscoelastic tests**	Not evaluated in COVID-19	X		X			X
	Lack of standardization between instruments and centers						
	Expensive						
**Thrombin generation assays**	Not evaluated in COVID-19	X					
	High sensitivity to heparins						
	Limited availability in clinical practice						
	Expensive						

(*) Heparin resistance can be due to HIT.

Note: the assessment of the in vivo effect of (any) anticoagulant treatment could be assisted with the monitoring of fibrin-related markers (D-dimers; fibrin monomers)

## Hemostasis assessment in the laboratory

COVID-19 is frequently associated with hemostasis disturbances (often referred to as “coagulopathy”, even if many if not all aspects of hemostasis can be affected), which significantly enhance the risk of thrombosis, and in particular venous thrombosis. Several studies have found an association between increased plasma D-dimers values and unfavorable prognosis in COVID-19 [1]. Different thresholds have been proposed for stratifying the risk of mortality (i.e. between 1000 or 3000 ng/mL [38, 39]), most often according to retrospective, methodologically weak studies, with limited statistical power. D-dimers at hospital admission could also be predictive of thromboembolic events [2, 11, 14, 40, 41]. However, in the presence of significant pulmonary inflammation, such as in severe COVID-19, fibrin deposits can occur within alveoli and pulmonary extravascular space, which has been confirmed in COVID-19 patients in autopsies series [42]. The lysis of those deposits could also contribute to the rise of D-dimers, which are thus not specific of intravascular fibrin formation [43, 44].

Nevertheless, the measurement of plasma D-dimers has been put forward by some expert groups as a laboratory criterion, along with clinical data, for stratifying COVID-19 patients according to their thrombotic risk, and to consider adjusted anticoagulation intensity. For example, the working group on perioperative hemostasis (GIHP stands for “Groupe d’Intérêt en Hémostase Périopératoire” in French) with the French group of studies on hemostasis and thrombosis (GFHT, stands for “Groupe Français d’Étude sur l’Hémostase et la Thrombose”) considered patients with plasma D-dimers levels > 3000 ng/mL as having a very high thromboembolic risk, and hence might benefit from increased doses of heparin [15]. In patients at high risk of thromboembolism, other authors also recommended continuing thromboprophylaxis after hospital discharge for a maximum of 45 days after individual assessment of the benefit/risk balance [45]. However, the variability between methods for measuring D-dimers raises questions about the use of a fixed threshold [46, 47]; an increased plasma D-dimers concentration with ongoing thromboprophylaxis should give consideration of the administration of higher doses of anticoagulants, and to the active search for thrombotic event.

The most seriously affected individuals (non-survivors and those admitted to intensive care), also have slightly longer PT than patients with more favorable prognosis [48, 49]. APTT is generally proportionally less prolonged than PT, probably due to an increase in plasma concentration of factor VIII, which is an acute phase reactant [48, 49]. A low platelet count at admission (i.e., < 200 × 10^9^/L), along with a further decline during hospital stay, has also been associated with increased risk of death [50–52].

In the majority of patients, hemostasis disorders associated with COVID-19 do not evolve towards disseminated intravascular coagulation (DIC), the diagnostic criteria of which include, according to the International Society on Thrombosis and Haemostasis (ISTH), a reduction in platelet count and fibrinogen levels associated with an increase in PT and markers of fibrin formation, or ‘fibrin-related markers’ (D-dimers or SFC, the former being the most used in Europe) [49, 53]. Among laboratory criteria, hypofibrinogenemia (< 1 g/L) is a late criterion and hence a poorly sensitive one, which can only be found in less than 50% of cases [54]. This observation can be explained by enhanced liver production of fibrinogen in response to systemic inflammatory response, which is hence effective to maintain normal plasma concentrations even in concomitance with consumption coagulopathy. A shortening of PT has also been reported in up to 30% of patients with COVID-19, which has been explained with increased fibrinogen concentration in plasma [55]. In this setting, the early identification of consumption coagulopathy can therefore be jeopardized, so that longitudinal monitoring of these parameters, at least in more severe patients admitted to the ICU, would be necessary to identify early changes that would indicate the onset of DIC.

Monitoring every 48 h the plasma concentration of PT, fibrinogen, D-dimers and platelet count has been proposed for continuously assessing the thrombotic risk and identifying timely alerts on possible venous thrombotic events, as reflected by markedly increasing concentrations of D-dimers within 24–48 h [15, 27]. An increase in the plasma D-dimers in patients with anticoagulant therapy may also warrant an increase in anticoagulation intensity, after weighting thrombotic and hemorrhagic risks, although there is no clinical evidence yet to support such D-dimers based dose adjustments.

However, the relevance of the utilization of D-dimers levels to tailor thromboprophylaxis in COVID-19 patients has been questioned, as plasma D-dimers levels could depend more on the lysis of extra-vascular rather than intra-vascular fibrin deposits [43, 44]. This would be consistent with the observation that SFC levels, which are thought to only depend on intra-vascular fibrin deposition, remain low in most COVID-19 patients despite high D-dimer levels [30]. Additional biomarkers of in vivo enhanced thrombin generation, such as SFC, may thus provide some additional useful information for managed care of COVID-19 [56]. SFC could also be an earlier marker of DIC than D-dimers, of which the concentration increases only after clot lysis has begun [56]. Measurement of plasma SFCs has therefore been proposed as an option besides that of D-dimers in the diagnostic scores for DIC proposed by the ISTH and the Japanese society of Thrombosis Hemostasis (JSTH) [57, 58]. However, the diagnostic thresholds vary considerably from one kit to another (two kits are available to the best of our knowledge: STA-Liatest FM® kit (Stago, Asnières-sur-Seine, France) and Nanopia SF (Sekisui Medical, Japan)), and there is a lack of extensive clinical validation [59, 60]. For instance, The STA-Liatest FM® kit (Stago, Asnières-sur-Seine) uses a monoclonal antibody (F405) specific for fibrin monomers. This antibody is produced using desAA-fibrin as immunogen in the presence of an anti-polymerization peptide (Gly-Pro-Arg-Pro, GPRP) [61]. Regarding desAA-fibrin, it is the result of an enzymatic cleavage of purified fibrinogen by the action of batroxobin (reptilase), with the release of fibrinopeptides A only; under those conditions, factor XIIIa cannot have any role, thus there is no ‘D-dimer’ motif. The evaluation of laboratory performance by an external quality control (EQA performed by ProBioQual) showed an inter-laboratory imprecision (coefficient of variation; CV) of ~ 30% for the three levels of controls tested. The half-life of SFC is slightly shorter than that of D-dimers (i.e., < 6 h), depending on the clinical context though (size and range of molecules produced, fibrinolytic activity and so forth) [62]. The half-life is hence longer than that of fibrinopeptides A and B (3–5 min), thrombin-antithrombin complexes (TAT; 10–15 min) and prothrombin fragments 1 + 2 (90 min) [63], so that is less dependent on the time of blood collection, considering that activation of coagulation cannot be a continuous, steady process. Nevertheless, the influence of the time elapsed between thrombosis onset, blood collection, and measurement, as well as the potential influence of anticoagulants remain to be studied [56]. For all assays, in particular for fibrinopeptides and TAT, the influence of preanalytical artifacts (thrombin formed during blood collection and processing) should be considered [56]. At this stage, additional evidence is needed to define the incremental clinical value of SFC over D-dimers in COVID-19 hemostasis monitoring.

### Limitations of measurement of the plasma concentration of D-dimers

Even if the usefulness of D-dimer testing in the management of COVID-19 patients remains uncertain, some expert groups suggested the use of this parameter together with clinical variables to stratify patients according to their thrombotic risk and determine thromboprophylaxis intensity accordingly [15–19]. This implies that test results shall be timely and reliable, and accurate threshold values need to be defined according to the specific clinical context and the immunoassay used.

**Fig. 1:**
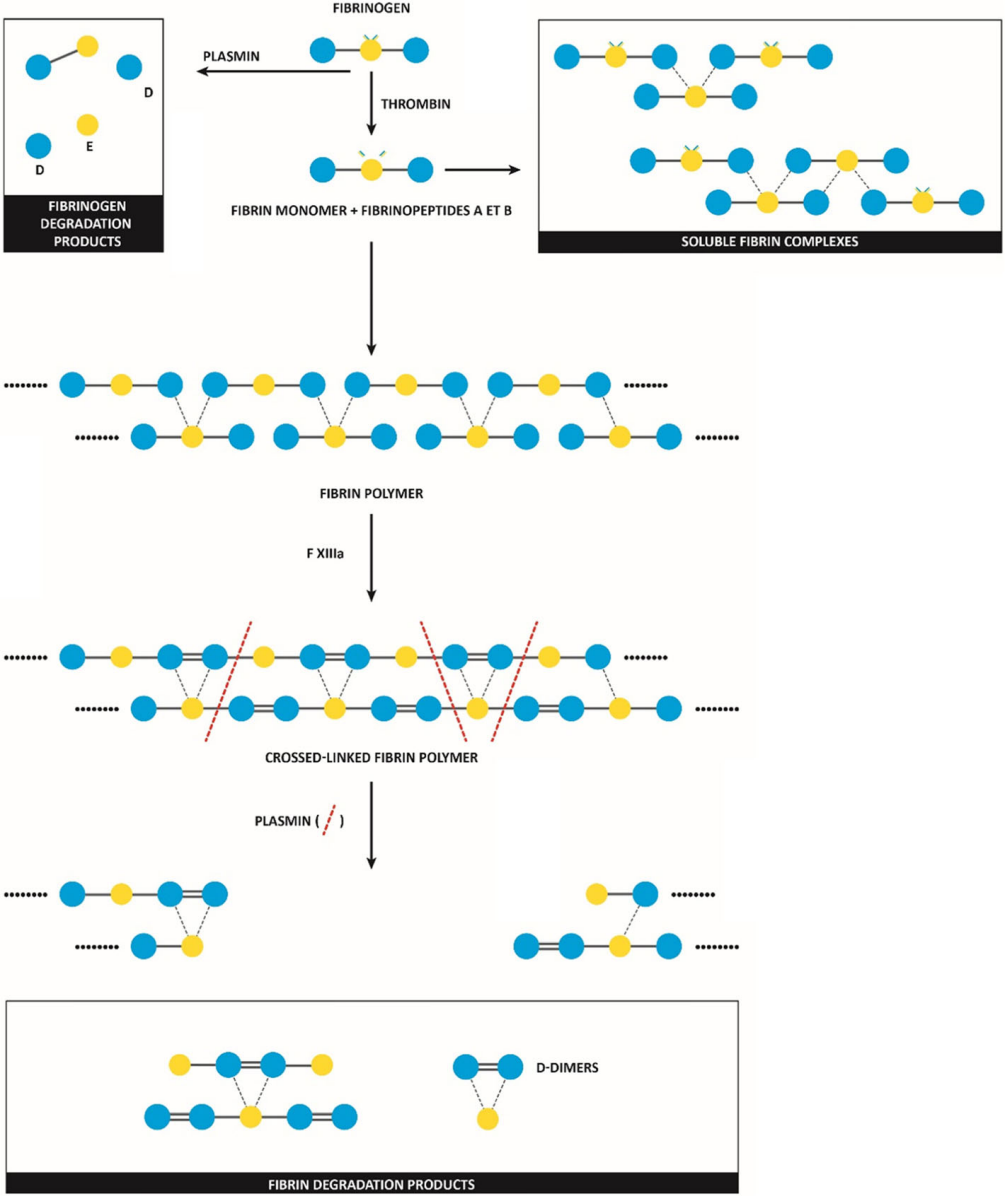
Mechanisms of production of: D-dimers, fibrin monomer, fibrinogen degradation products and fibrin degradation products. Fibrinogen is composed of two lateral regions “D” and one central region “E” connected by coiled coils: the formula is D-E-D. Fibrinopeptides A and B located at the N-termini of A-alpha and B-beta chains (× 2) on the E region are cleaved from fibrinogen by thrombin, resulting in the production of a fibrin monomer (FM). FMs are highly reactive and if locally formed and concentrated, quickly interact with one another by to form ​a two-stranded fibrin polymer. These polymers then aggregate laterally to make fibers (not shown). Activated factor XIII covalently crosslinks adjacent D regions (belonging to two fibrin monomers), which tightens the fibrin strand, increases clot stiffness, and makes it more resistant to degradation by plasmin. Other crosslinks also occur, not shown here for simplicity’s sake. The physical state is a gel - such polymerized structures are no longer soluble. During fibrinolysis, plasmin can cleave fibrin polymers between adjacent D and E regions, but cannot separate covalently linked D regions. This produces fibrin degradation products of different size, containing the ‘D-dimer’ motif, and when small enough are soluble. FM can escape in the fluid plasma phase, the more so if formed in a disseminated manner (systemic thrombin generation), and then quickly binds to fibrinogen molecules, or fibrinogen degradation products, bringing the polymerization process to an end; they hence remain soluble because they are small enough. These compounds are known as ‘soluble fibrin complexes’ (SFC). In the presence of hyperfibrinolysis (systemic, disseminated), PAI-1 (plasminogen activator inhibitor) and alpha2-antiplasmin can be overwhelmed, and uninhibited​ plasmin can diffuse in the plasma fluid phase; under those conditions, plasmin can also cleave fibrinogen molecules, resulting in fibrinogen degradation products production. ‘FDP’ may refer to both fibrinogen and fibrin degradation products

Soluble fibrin degradation products containing the D-dimer motif constitute a heterogeneous set of molecules produced during degradation of polymerized fibrin network, which has been previously covalently cross-linked by activated factor XIII. This explains differences observed between fibrinogen and fibrin degradation products (FDP), the former being generated by degradation of fibrinogen from the fibrinolytic system (Fig. 1) [58]. A large inter-laboratory variability has been reported for the measurement of the plasma D-dimers concentration, which mostly reflects the different assay methodologies, the different mix of antibodies with variable antigenic specificity, the individual calibration and the variation of measuring units [64–66]. The lack of internationally certified calibrators and quality controls also challenges to achieve better degree of universal harmonization. The large inter-individual variability (depending among others on renal function), is a further source of uncertainty in test results interpretation. A diagnostic threshold that has been validated within a specific clinical setting, using a certain assay, cannot be translated to different analytical conditions and different clinical settings [67]. The diagnostic thresholds shall hence be validated according to the method used and for the intended diagnostic purpose. Most of the available D-dimers assays have also been developed to have the best reproducibility around the threshold value used for excluding deep vein thrombosis and/or pulmonary embolism, which is usually around 500 ng/mL. Therefore, their performance at higher values, such as those proposed for initiating high dose anticoagulation in COVID-19 patients (i.e., over 3000 ng/mL), is probably suboptimal and would need to be assessed in order to avoid the use of inaccurate results in these patients. For example, external quality controls performed with moderately elevated D-dimers samples (target value 4000 ng/mL FEU) identified method-specific D-dimers means ranging from 470 to 10,150 ng/mL FEU (all methods coefficient of variation: 23%) [68]. As a possible solution, the threshold values of plasma D-dimers could be adjusted based on assay methodology. For example, for DIC diagnosis according to the ISTH definition, the appropriate D-dimer cut-off value for 2 points ranged from 3500 ng/mL to 6500 ng/mL, depending on the reagents used [69].

The potential impact of many preanalytical variables on measurement of plasma D-dimers should also be considered, as this test is vulnerable to the excessive presence of free hemoglobin in plasma, and becomes virtually uninterpretable when the plasma free hemoglobin concentration is over 30 g/L [70]. Hyperlipemia, hyperbilirubinemia or a high concentration of immunoglobulins can also generate a bias in the measurement, though the influence of these conditions has been less studied [70]. However, it has to be considered in the context of COVID-19 due to the high concentration of immunoglobulins measured in some of these patients 2 weeks after the symptom onset [71].

### Laboratory investigation of fibrinolysis

Many of the methods available for the study of fibrinolysis have significant drawbacks. Fibrinolysis assays are technically challenging, time consuming and cannot be easily automated, thus explaining their underuse in clinical laboratories. Under physiological circumstances, fibrinolysis takes many hours or days to develop in healthy blood after clotting, and this is indeed a major limitation for rapidly assessing the ‘global fibrinolysis capacity’ (GFC). The accelerated (albeit remaining long) assessment of fibrinolysis needs prior removal of inhibitors or the addition of tPA to initiate plasmin generation, complicating our appraisal of what is going on in vivo. Plasma-based systems, for example, where clotting and lysis may be easily monitored with turbidimetric assessment, need tPA to accelerate the reaction [72]. Alternatively, euglobulin may be prepared from plasma, thus lowering the concentration of fibrinolytic inhibitors (including PAI-1 and antiplasmin) [73, 74].

Individual components of the fibrinolytic system can also be measured. Although interesting evidence is emerging especially for PAI-1 in COVID-19 [30], its measurement is limited by large inter-laboratory variability, which is attributable to the different assay methodology, antigenic specificity of the antibodies, the lack of internationally certified calibrators and quality controls, as well as to the many measuring units that can be used for reporting data.

Interestingly, PAI-1 follows a circadian rhythm in humans, with a morning peak (around 8 AM) independent from the sleep pattern [75, 76]. The time-interval between blood collection and analysis, the anticoagulant used in sample tubes and storage temperature are other variables that are known to have an impact on plasma PAI-1 measurement [77–79].

A weak stability at room temperature of the plasma used for assessing fibrinolysis (and especially PAI-1) has been occasionally described, whilst hemolysis (with release of cell free hemoglobin and other intracellular components) may contribute to produce an inhibitory effect on some fibrinolysis assays [80]. The contact phase (i.e., activated factor XII) also stimulates fibrinolysis through conversion of single chain urokinase-type plasminogen activator (scu-PA) in uPA [81]. Therefore, standardization of preanalytical and analytical steps is of utmost importance for obtaining accurate and reliable results of most fibrinolysis assays.

Regarding viscoelastic tests, ROTEM and TEG currently lack sensitivity to disordered fibrinolysis but there is still room for improvement [82].

As some extracellular vesicles are able to facilitate plasmin generation, it would be interesting to assess the contribution of such vesicle-dependent fibrinolytic activity in COVID-19 [83, 84].

### Viscoelastic tests

Viscoelastic tests (VETs) are “global” hemostasis tests evaluating mechanical properties of the clot as it forms and lyses. The most frequent used VETs comprise thromboelastography (TEG) and rotational thromboelastometry (ROTEM). These commercial assays are embedded in the care for critically ill patients in many institutions, in particular for guiding transfusion management in patients at high risk of bleeding. In general, these assays are sensitive to detect hypocoagulability related to consumption of coagulation factors like fibrinogen, but hypercoagulability can also be detected. Indeed, increased clot firmness has been associated with occurrence of venous thromboembolism (VTE) in various clinical settings [85–91], and has hence been proposed for evaluating the thrombotic risk associated with SARS-CoV-2 infection.

In COVID-19 patients, whole blood rotational thromboelastometry (ROTEM) has been able to detect accelerated clot formation and increased clot strength, persisting for at least 5 days [92]. Similar findings were reported by other groups using ROTEM and Quantra instruments, with both elevated platelets and fibrinogen contribution to clot strength [93, 94]. Increased heparin prophylaxis dose was associated with attenuation of fibrinogen contribution to clot strength and fibrinogen level, although lacking control conditions, this may have been a chance finding. Data regarding fibrinolysis evaluation in COVID-19 using VETs is sparser; hypofibrinolysis has been identified in one study [95], although another could not confirm this result [92]. Only one study evaluated the association between clinical outcomes and VETs parameters in COVID-19. The authors identified that the lack of clot lysis at 30 min on citrated kaolin TEG with heparinase was associated with VTE, whereas increased clot strength was not [95]. The combination of absence of lysis at 30 min with D-dimers levels > 2600 ng/mL was also strongly associated with VTE and with the need for dialysis [95]. Nonetheless, the place of VETs in the management of COVID-19 deserves further evaluation.

When using VETs, some drawbacks have to be considered. First, these tests are generally poorly sensitive to platelet function and mild fibrinolysis disorders [96, 97], and their sensitivity to fibrinogen levels is quite variable depending on the test methodology [98]. Second, even if considered as being global tests, they do not evaluate the contribution of endothelium, whose dysfunction likely contributes to COVID-19 associated hemostasis disturbances [34]. Moreover, similarly to D-dimers and SFC, correlation between methods is moderate, and inter-laboratory variation is high [99], but probably improving with introduction of new methods (ROTEM Sigma®, TEG6S® and Quantra®). Regarding preanalytical conditions, the time-interval between blood collection and VET [100, 101], anticoagulants and/or additives used in sample tubes [102, 103], over or underfilling of blood tubes [104], hemolysis [105] and hematocrit [106–108] can influence the test results. Pneumatic tubes transport systems (PTS) may also exert little influence on test results, depending on acceleration forces of the local system [109–112]. Therefore, this effect should be evaluated locally when utilization of PTS is considered for blood sampled for VETs. Finally, the accuracy of these tests for bleeding management or thrombotic risk stratification has not been validated in any hyperinflammatory context. Caution is therefore required when using VETs in COVID-19 patients, and further studies are needed to precise their added value in the management of these patients.

### Thrombin generation assay

Thrombin generation assays (TGA) enable an integrative approach of coagulation. Commercial assays include Technothrombin® TGA kit from Technoclone and Calibrated Automated Thrombogram® (CAT) and ST-Genesia® from Stago Diagnostica [113, 114]. While TGA may be used to detect hypercoagulability, the frequent use of heparin/low molecular weight heparin (LMWH) in hospitalized COVID-19 patients could make this test relatively unsuitable for evaluating hypercoagulability. Modification of the TGA conditions, i.e. by adding polybrene or heparinase as neutralizing agent [115], could be of interest to eliminate heparin. However, data has not been reported yet in COVID-19 patients. Of note, pre-analytical variables and choice of the reference plasma to normalize the result also have important impact on TGA measurement and must be controlled in order to reduce the variability of measurement [116].

## **Anticoagulation -** laboratory monitoring

Due to the high thrombotic risk associated with this infection, systematic antithrombotic prophylaxis should be administered and to date, parenteral administration of a heparin preparation is the preferred procedure in the hospital setting.

### Choice of anticoagulant

LMWH is recommended as first-line therapy for the prophylaxis of VTE in hospitalized COVID-19 patients [15, 20, 117, 118]. Direct oral anticoagulants (DOACs) or vitamin K antagonists, are not recommended because of the risk of drug interactions, among others with some antiviral drugs, the expected broad fluctuation in plasma concentrations (for DOACs), especially in patients at higher risk of rapid clinical deterioration, and because of the late onset of anticoagulation and higher risk of bleeding with VKA [119]. Heparin, and especially the long heparin chains, was reported to also exhibit anti-inflammatory activity in addition to the anticoagulant effect, by binding and neutralizing inflammatory cytokines and acute phase proteins, while potentially exerting a protective effect on endothelium [120]. It also interferes with neutrophils recruitment into tissue and impairs neutrophil function by inhibiting the activity of the neutrophil protease’s human leukocyte elastase and cathepsin G, which can promote inflammation [121, 122]. It has also been hypothesized that heparin may hinder the interaction between the virus and the host cell through non-specific ionic bond, and thus may contribute to decrease the rate of infected cells [120, 123]. However, we do not yet know precisely to what extent heparin is clinically effective in this infection.

Compared to UFH, LMWH has better bioavailability after subcutaneous administration and longer duration of action, allowing daily administration in one or two injections. No regular laboratory monitoring is necessary for treatment with LMWH because of the predictable anticoagulant activity after administration of doses adjusted to body weight on the one hand, and the lack of formal association demonstrated between laboratory tests results and clinical efficacy or complications on the other [124]. However, a single anti-Xa activity measurement can be proposed in case of administration of high or intermediate doses in a patient with moderate renal impairment, with a BMI < 18 kg/m^2^ or > 30 kg/m^2^, or during pregnancy, mainly to rule out drug accumulation [125]. In the event of increased doses in patients at enhanced thrombotic risk, measurement of anti-Xa activity is also recommended 4 h after the third dose to exclude accumulation. The anti-Xa value must be based on a calibration curve with the specific heparin type and interpretation of test result must also be made for the same compound; e.g. with therapeutic doses of enoxaparin, the mean peak concentration observed is 1.2 IU/mL. This measurement can be repeated for example in case of renal function impairment.

UFH is only recommended in case of severe renal failure (creatinine clearance according to Cockcroft-Gault equation < 30 mL/min), extracorporeal membrane oxygenation (ECMO) [15] or significant bleeding risk (shorter half-life than LMWHs, easier neutralization by protamine) [126]. The risk of heparin-induced thrombocytopenia (HIT) is also much higher with UFH [127]. Regular laboratory monitoring during anticoagulation is necessary because of the high inter- and intra-individual variability in the anticoagulant response [125].

### Laboratory monitoring of unfractionated heparin treatment

Adjustment is thought to be required because of high inter and intra-individual variability. Historically, adjustment of UFH dosage was based on APTT. The measurement is performed 4 to 6 h after initiation and any dose change, and once a day at least, to reach a target APTT ratio between 1.5 and 2.5. This therapeutic target dates from work in 1972 and has never been confirmed in large clinical studies [128]. Since then, the number of reagents used for the APTT has exponentially increased, the sensitivity of which is very different both to heparin and biological interference (including several proteins of the acute phase) [129–131]. Therefore, calculation of the APTT ratio corresponding to an anti-Xa activity between 0.3 and 0.7 IU/mL would be recommended for each analyzer and each new batch of reagents. This calculation should best be performed with plasma samples from patients treated with UFH because the use of plasma spiked with UFH gives less relevant results due to the influence of the metabolism of heparin in vivo and its bioavailability [132]. For some reagents, the relationship between heparin levels and APTT is linear, but this can change in case of significant inflammation [133]. Most importantly, APTT is very dependent on pre-analytical conditions. Among others, platelet factor 4 (PF4) released by activated platelets during inadequate sampling procedure or prolonged delay before centrifugation can neutralize part of the heparin, leading to a risk of underestimating its activity [134].

Several biological parameters can cause a prolongation of the APTT (e.g. high CRP, presence of lupus anticoagulants, coagulation factors deficiency, high plasma concentration in FDPs which oppose the polymerization of fibrin) or its shortening (e.g. increased plasma concentration of acute phase proteins such as FVIII and fibrinogen) [63, 135, 136]. The influence of these parameters will also depend on assay methodology, the type of reagents, and has a high inter-individual and intra-individual (i.e., during hospitalization) variability [137, 138]. For these reasons, the GFHT advises against the use of APTT for monitoring treatment with UFH [133]. Moreover, the use of APTT is problematic when this test is prolonged prior to initiation of UFH treatment (for example, in case of lupus anticoagulants or coagulation factors deficiency); anti-Xa target should also consider the etiology of this prolongation when clinically relevant (i.e. defects with a bleeding risk).

In COVID-19, the increased plasma concentrations of fibrinogen and FVIII can cause a shortening of APTT (observed in 16% of affected patients) [55], and this may lead to underestimating the anticoagulant effect of heparin. This situation can contribute to excess heparin dosing, enhancing the bleeding risk, and underlines the importance of collecting a basal APTT value before anticoagulation. This can be problematic in some settings, e.g. in the ICU, where patients are frequently transferred with anticoagulation already started at intermediate or therapeutic doses [13]. Conversely, APTT prolongation may be related to the transient increased levels of antiphospholipid antibodies, a situation encountered during viral infections [139], including COVID-19 [10, 140–143], or when CRP is high [138], thus leading to a risk of heparin underdosage. Lupus anticoagulant screening can also be falsely positive in the presence of variables prolonging clotting time of tests used for its screening (for example, elevated CRP or presence of anticoagulants, among which heparin) [143–146]. The APTT may also be prolonged in the presence of DIC (which is relatively rare in COVID-19). In this situation, its measurement by means of an optical system may become uninterpretable: an immediate and gradual decrease in light transmittance can be observed even before clot formation due to the presence of a complex between CRP and very low density lipoproteins in the presence of calcium [147], thus rendering the measurement unreliable [148]. Therefore, mechanical methods are advisable in this circumstance, where measurement anti-Xa activity may even be the best choice.

Thus, heparin monitoring with aPTT may be challenging in COVID-19 patients due to the hyper-inflammatory status of the patient. Indeed, the high fibrinogen and factor VIII levels, the interference of CRP (depending on the reagents used) and also the potential presence of antiphospholipid antibodies may affect the aPTT. Therefore, anti-Xa activity seems more suitable to monitor UFH treatment in these patients and more generally in ICU patients for the very same reasons. However, there are several caveats here as well. First, FXa is not the essential target of UFH. Its inhibition is studied under very artificial conditions: in the fluid phase (and not within the prothrombinase complex formed on a phospholipid surface) and in a calcium-depleted medium. The in vivo inhibitory activity of UFH is indeed three times stronger towards FIIa than FXa [149]. This difference is further artificially increased in vitro by use of low calcium concentrations in the assay mixture: the anti-Xa activity was halved under these conditions, compared to physiological concentrations of calcium, but the effect of low calcium on anti-IIa activity is more limited [149]. Having mentioned that, a good correlation exists in vitro between anti-Xa and anti-IIa activities, thus enabling the use of the former test to assess the effect of heparin therapy. The anti-Xa assay consists of measuring in vitro the residual activity on a chromogenic substrate specific for FXa added to citrated plasma. Compared to APTT, this test has the advantage of being less vulnerable to biological interference (possible interference of free hemoglobin and bilirubin in case of significant elevation [150]) and less dependent on pre-analytical conditions - with the notable exception of PF4 released in vitro by platelets. Even if the validity of anti-Xa activity of UFH in the presence of an important inflammatory syndrome has not been formally established (or for that matter under any circumstances), this measure will be less impacted in this context, particularly if the reagents contain antithrombin (AT). However, it is not advisable to use kits with exogenous AT to avoid overestimation of anticoagulant activity in case of AT deficiency, with risk of heparin underdosing. The plasma concentration of AT can indeed decrease in case of sepsis [151]. Only few data are available on the sensitivity of kits without exogenous AT to changes in plasma AT concentration [152]. Some reagents also contain dextran sulfate, which will displace heparin from its non-specific binding (including PF4). Particularly, the influence of PF4 released by activated platelets is therefore minimized, which is favorable for limiting the impact of pre-analytical conditions on test result, but problematic when the concentration of PF4 is actually high in vivo. Unlike a ‘global’ test like APTT, the measurement of anti-Xa activity is insensitive to fluctuations in the underlying hemostatic state (for example, coagulation factor defect following hemorrhage or DIC), which should prompt an adjustment in therapeutic targets to the clinical context and the possible identification of such defects. Finally, significant variability in heparin sensitivity has been reported between the different commercial kits [153–156].

The therapeutic range in terms of anti-Xa activity is considered between 0.3 and 0.7 IU/mL [125]. These values ​​are derived from the work of 1972 that was previously mentioned. It was hence inferred from the APTT target to be reached for secondary prevention of VTE. The bottom line is that it lacks validation for the same reasons [157]. The application and interpretation of these tests in a hyperinflammatory context also raises important questions. Given the very high thrombotic risk described or suspected in some subgroups of patients, the GIHP suggested narrowing the target of anti-Xa activity in the upper zone, to 0.5–0.7 IU/mL, for those at the highest thromboembolic risk [15]. This increase in dosage, not supported by objective data, remains debated [20–23], whilst ongoing prospective, multicenter clinical trials aim at addressing this question. In addition to the selection of patients who could potentially benefit from increased doses of anticoagulants, the question of treatment duration is a major one. The resolution of the inflammatory syndrome should be accompanied by a reduction in the thrombotic risk, thus exposing the patient to excessive anticoagulation and risk of bleeding when higher doses of heparin are maintained. However, no firm recommendation on duration and intensity of COVID-19 anticoagulation can be made at this time.

In patients receiving UFH, a laboratory resistance to the anticoagulant effect of heparin, arbitrarily defined by failure to reach the therapeutic target despite the administration of doses > 1.5 times usual doses, which are about 400 to 600 U/kg/24 h [158, 159], is frequently observed in COVID-19 [13, 160], and adds to the clinical resistance previously outlined (occurrence of thrombotic events under well-conducted drug thromboprophylaxis). The hyperinflammatory context could also explain part of this observation. Indeed, UFH is able to non-specifically bind several acute phase proteins as well as activated endothelial cells and platelets, thus limiting its anticoagulant activity [161, 162]. The administration of an initial bolus of UFH is therefore needed to saturate non-specific fixation [163]. The increased plasma concentration of fibrinogen and FVIII will also contribute to generate heparin resistance when the effect is monitored with the APTT, which is less likely to be observed when anti-Xa activity is used. Acquired AT deficiency by consumption or production defects (negative protein of the acute phase) could also contribute to the heparin resistance observed in some COVID-19 patients [151], especially those most seriously affected [10, 49], but in most patients it does not justify the prescription of AT concentrates. To the best of our knowledge there is one single prospective interventional study on UFH monitoring in case of laboratory heparin resistance. In this study, the utilization of anti-Xa activity instead of aPTT permitted to avoid UFH dosage escalation with similar clinical outcomes [164]. Whether this holds true for COVID-19 patients remains to be established. When heparin resistance is suspected based on APTT values, UFH shall be administered according to the anti-Xa activity [165]. The advantages and limitations of APTT vs anti-Xa for UFH monitoring are summarized in Table 3.

**Table 3:** Main advantages and limitations of APTT and anti-Xa activity for UFH treatment laboratory monitoring

	Advantages	Limits
**APTT**	- largely available, low cost - sensitive to clinically relevant changes of coagulation (coagulation proteins increases or deficiencies)	- numerous interferences; optical methods can be unreliable in case of DIC
		- heparin sensitivity is highly reagent dependent
		- APTT prolongation target needs to be established for each new batch of reagents
		- APTT before UFH needed
		- clinically irrelevant changes, or of dubious clinical relevance
		APTT prolonged with:
		• presence of antiphospholipid antibodies (viral infections)
		• high CRP (depending on the reagent)
		• high plasma levels in FDPs
		• preanalytical (e.g., heparin/EDTA contamination, under-filling, delayed centrifugation, hypertriglyceridemia, hyperbilirubinemia)
		APTT shortened with:
		• preanalytical (e.g., prolonged venous stasis, vigorous mixing, coagulation of the sample, PF4)
		• high factor VIII levels
**Anti-Xa activity**	- less vulnerable to biological interferences - no requirement for measurement before UFH administration	- preanalytical interferences: PF4*; free hemoglobin and bilirubin if significant elevation
		- insensitive to fluctuations in the underlying coagulation state (i.e., coagulation factor increases or defects), of potential clinical relevance
		- AT deficiency (e.g. in sepsis): risk of heparin underdosing with kits containing exogenous AT; sensitivity to endogenous AT not evaluated with kits that do not contain exogenous AT
		- variability in reagents composition (AT, dextran…)
		- therapeutic range poorly defined
		- not validated in hyperinflammatory states
		- less available, more expensive

*PF4 released by activated platelets during poor sampling technique will neutralize UFH, leading to an underestimation of its activity

*AT* antithrombin, *APTT* activated partial thromboplastin time, *UFH* unfractionated heparin, *PF4* platelet factor 4; *CRP* C-reactive protein, *DIC* disseminated intravascular coagulation, *FDP* fibrinogen and fibrin degradation products; *FVIII* factor VIII.

### Diagnosis of heparin-induced thrombocytopenia (HIT)

A final aspect of heparin monitoring is screening for HIT. A platelet count should be performed before administering the first injection of heparin, if possible, or as soon as possible thereafter. In the COVID-19 setting, it is reasonable to monitor the platelet count regularly between the 4th and the 14th day following the initiation of heparin therapy (once or twice a week in case of LMWH treatment, two to three times a week during UFH treatment), then once a week until the end of the first month of therapy. The development of thrombocytopenia (< 100 × 10^9^/L) or the rapid decrease in platelet count (especially if ≥50% in less than 24 h) should then suggest the diagnosis of HIT [166]. However, especially in the presence of acute inflammation and infection, other etiologies may explain a decrease in platelet count. Therefore, a systematic evaluation of clinical probability of diagnosis allows better identification of patients in whom the occurrence of this complication must be suspected, and for whom laboratory work-up for HIT antibodies is indicated. This evaluation is generally performed with the 4Ts’ score, much studied [167, 168] [159], despite its limitations, particularly in more complex situations such as those encountered in ICU (no consensus on the drugs responsible for thrombocytopenia, many other causes of thrombocytopenia, very high negative predictive value but not absolute (e.g. thrombosis in the absence of thrombocytopenia), insufficient data on platelet count, weak agreement in the determination of the 4th criteria (other causes of thrombocytopenia)) [166, 169, 170].

In case of strong suspicion, or as soon as the antibodies are identified, treatment with heparin should be stopped and replaced by a direct thrombin inhibitor (DTI; argatroban, bivalirudin) or by danaparoid sodium. Of note, the presence of a DTI can lead to underestimation of plasma fibrinogen concentrations by inhibition of the thrombin present in Clauss’ reagent [171]. This interference will vary depending on the thrombin concentration used in the reagent [172, 173]. To a lesser extent, interference may also exist in the presence of high concentration of UFH (0.6 to 2.0 IU/mL, depending on the reagent), which would exceed the neutralization capacities of the reagent used, or in the presence of high concentration of FDPs (> 100–130 μg/mL) [173].

## Conclusion

SARS-CoV-2 (COVID-19) infection is associated with a laboratory prothrombotic state and a high incidence of thrombosis. The follow-up of COVID-19 patients by hemostasis testing could be pivotal, both in terms of risk evaluation and therapeutic monitoring, though the limitations of the tests used must always be acknowledged. Longitudinal studies are needed to clarify which parameters are the most relevant in terms of thrombotic risk assessment and how to use them for patients’ management (clinical implications, optimal cut-offs, frequency of measurement, etc.). Measuring anti-Xa activity is recommended to guide UFH treatment, although this assay is not without drawbacks. Whichever the test used, the attitude adopted must fit local analytical conditions. Additional studies are needed to gain knowledge on the complex, variable and changing disturbances of hemostasis in COVID-19 patients and its interactions with the proinflammatory and infectious status of these patients.
